# Evolutionary Ecology of Fish Venom: Adaptations and Consequences of Evolving a Venom System

**DOI:** 10.3390/toxins11020060

**Published:** 2019-01-22

**Authors:** Richard J. Harris, Ronald A. Jenner

**Affiliations:** 1 Venom Evolution Lab, School of Biological Sciences, The University of Queensland, St Lucia, Brisbane, Queensland 4072, Australia; 2 Department of Life Sciences, the Natural History Museum, Cromwell Road, London SW7 5BD, UK

**Keywords:** evolution, ecology, fish, venom, evolutionary ecology, coevolution, natural enemy interactions, ecological niche, aposematism, mimicry

## Abstract

Research on venomous animals has mainly focused on the molecular, biochemical, and pharmacological aspects of venom toxins. However, it is the relatively neglected broader study of evolutionary ecology that is crucial for understanding the biological relevance of venom systems. As fish have convergently evolved venom systems multiple times, it makes them ideal organisms to investigate the evolutionary ecology of venom on a broader scale. This review outlines what is known about how fish venom systems evolved as a result of natural enemy interactions and about the ecological consequences of evolving a venom system. This review will show how research on the evolutionary ecology of venom in fish can aid in understanding the evolutionary ecology of animal venoms more generally. Further, understanding these broad ecological questions can shed more light on the other areas of toxinology, with applications across multiple disciplinary fields.

## 1. Introduction

Animal venoms have been the subject of much research, particularly in reptiles, arachnids, insects, and cone snails [1,2,3,4,5,6]. The biological activity of venom components and their characterisation have been at the forefront of toxinology for many years. This has led to some ground-breaking biochemical, genetic, evolutionary, and pharmacological discoveries [7,8,9,10]. In contrast, the evolutionary aspects of venom, particularly their evolutionary ecology, remain poorly studied. Investigating the evolution of venomous traits in the context of their ecology can answer many questions about how and why venom systems have evolved and shed light on the adaptive value and ecological implications of evolving a venom system. Venom systems play a vital role as key fitness components that facilitate survival and reproductive success. Yet, traits relevant to understanding the evolutionary ecology of venom systems, including their morphology, the behaviour of venomous organisms, the presence and nature of aposematic, and mimetic traits, as well as the niche spaces and life histories of venomous species all deserve more detailed investigations across a broader range of venomous species.

Venomous fish have been relatively poorly studied, both with respect to the composition and evolution of their venoms [11,12,13]. The focus of this review is to summarize what is known about the evolution of fish venoms considered from an ecological perspective and, where possible, to place it in the context of the evolutionary ecology of other venomous/toxic taxa.

### A Brief Introduction to Venomous Fish: Morphology and Biochemistry

**Figure 1:**
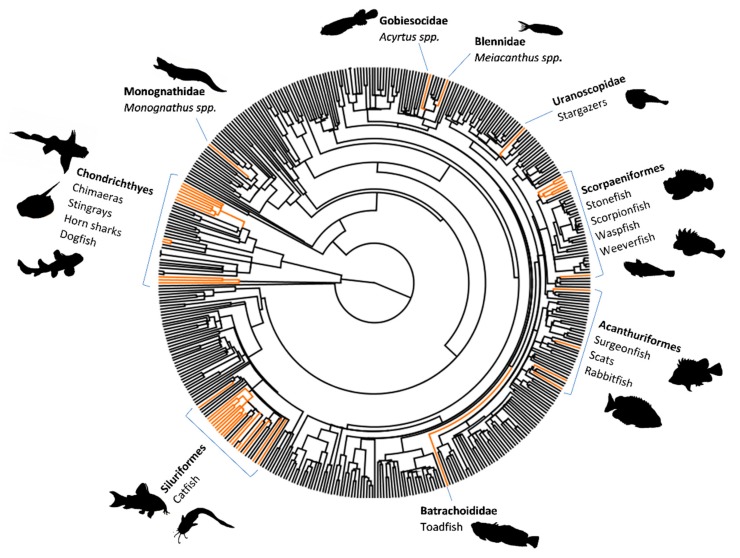
A phylogenetic tree of fish families (excluding cyclostomes) highlighting all known venomous families in orange. The exact number of venomous fish families lies between 58 and 63 [12]. The phylogenies of Chondrichthyes and Actinopterygii were obtained from the Time Tree web project (www.timetree.org) and merged using R package phytools [16].

Fish venom systems are thought to have convergently evolved 19 times (we include the toxic buccal secretions of lamprey as venom) (Figure 1), with more than 2900 species utilising venom as a form of defence, while a few species use venom for predation/competition [12,13,14,15]. Venom evolution has had a particularly large impact in the lineages Scorpaeniformes (scorpionfish and relatives) and Siluriformes (catfish), which together comprise the majority of venomous fish species [12] (Figure 1).

**Figure 2:**
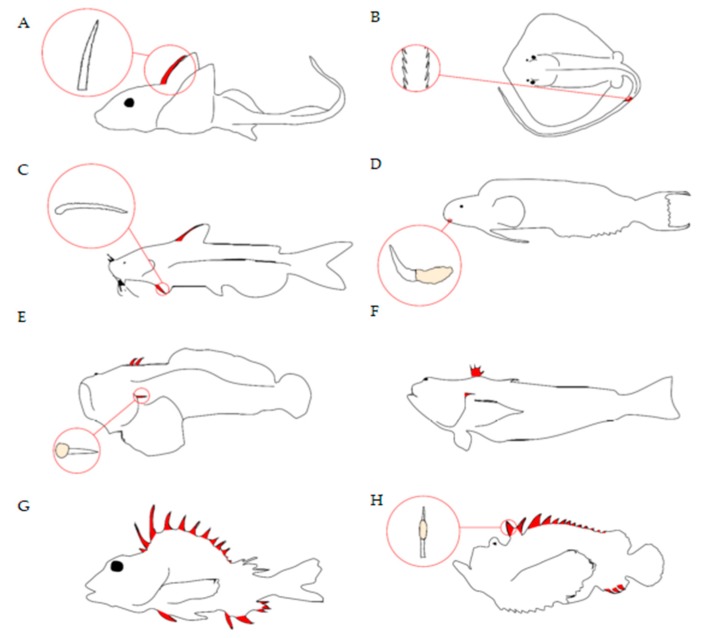
Examples of the different morphological structures used by fish to deliver venom. This figure is reproduced from Ziegman and Alewood [11], 2015, MDPI. The venom apparatuses are highlighted in red: (**A**) Serrated dorsal spine of chimera; (**B**) serrated caudal spine of stingray; (**C**) serrated pectoral spine of catfish; (**D**) canine tooth of fangblenny; (**E**) dorsal and opercular spines of toadfish; (**F**) dorsal and opercular spines of weeverfish; (**G**) dorsal, pectoral, and pelvic spines of gurnard perch; and (**H**) dorsal and anal spines with venom gland in stonefish.

Fish deliver venom through a range of structures, such as spines, barbs, and teeth/fangs [12,13] (Figure 2). Venom spines can be part of dorsal, pectoral, pelvic, and anal fins, as well associated with or positioned close to the operculum, whilst stingray barbs are found on the tail. Morphological analyses of fish venom systems indicate that spines are usually associated with some form of venom gland/secretory cells located near or surrounding the spines. Spines usually contain an anterolateral groove that allows venom to move from the base of the spine to the tip in a hypodermic fashion [12,13], allowing the toxins entry into the envenomated target via a wound. Although this is the general morphology of venom spines, there are differences between species (Figure 2) as would be expected for convergently evolved systems (see Smith et al. [12] for a more in-depth review).

Fish venoms contain a plethora of compounds, the majority of which are defensive in nature, with the sole purpose of causing discomfort and pain to any potential predator. Bioactive toxins from fish venoms cause a range of neuromuscular, cardiovascular, cytotoxic, and nociceptive effects [11]. The biochemistry of toxins also differs between and within lineages. Large pore-forming toxins, such as stonustoxin and verrucotoxin can be found in many scorpaenid fish (e.g., *Synanceia horrida* and *S. verrucosa*). Whilst there are many other protein/peptide toxins (dracotoxin, trachinine, and nattectin, etc.) that have been isolated from different species (e.g., *Echiichthys vipera, Trachinus draco,* and *Thalassophryne nattereri*), they are generally poorly studied [11]. Toxins with unusual and unique effects have also recently been discovered in some species (*Meiacanthus grammistes*) [17]. The proteomic analysis of fish venom is challenging due to the difficulty of collecting them as well as their lability and the problems of avoiding contamination of the venom samples with mucus from the body surface [18,19]. Therefore, efforts have been made to develop better methods of proteomic analysis of these delicate venom molecules for characterisation and functional analysis [20,21].

## 2. Evolving A Venom System

### 2.1. The Basics of Antagonistic Coevolution: The Classic “Arms Race”

Before discussing the evolutionary ecology of fish venom systems, we need to clarify some of the main concepts in this field. Antagonistic coevolution is arguably the main driver in the evolution of defensive systems [22,23]. Three main ecological functions of venom have been identified: defence, predation, and competition [2]. Antagonistic coevolution is the key to understanding how predator–prey and host–parasite interactions have led to the defensive function of venom, and it provides the context within which reciprocal selection pressures create a constant evolutionary “arms race” [22,23,24,25]. Venom is one of the most conspicuous and most frequently evolved adaptations thought to have evolved via such arms races. Despite this, there is still little empirical evidence supporting the impacts of these arms races on venom evolution. Moreover, recent studies have indicated the existence of other kinds of coevolutionary dynamics, such as phenotype matching between venomous predator and prey or local adaptation of predatory venom eliciting no detectable coevolutionary response in prey [26,27].

Prey can respond in two ways to these predatory pressures; they can evolve avoidance strategies and/or evolve antipredator defences [28]. Thus, defensive venoms can evolve in response to intense predator interactions with prey. It is useful to think of these strategies in terms of Endler’s (1986) five stages of predation: detection, identification, approach, subjugation, and consumption. Antipredator defences have evolved to disrupt each of these stages of predation. The primary stages of prey detection, identification, and predator approach have led to such evolutionary defences as camouflage and crypsis. The secondary stages of predation, prey subjugation and consumption, have led to the evolution of chemical defences, such as toxungens, poisons, and venoms [29]. The evolution of defensive squirting or spitting of venom in several lineages, such as snakes, scorpions, hymenopterans, and assassin bugs, also plays a role in deterring predator approach once the animal has been detected. The possession of these powerful chemical weapons consequently led to the evolution of aposematism and mimicry. Once evolved, aposematism and mimicry could then disrupt the earlier stages of predation and help minimise the use of metabolically costly venom toxins. However, aposematism and mimicry would not evolve without effective defensive mechanisms, such as venom, being in place. These toxic defences are commonly a last resort strategy, with chemically defended organisms usually having some other and cheaper form of defence to deter the early stages of predation, e.g., camouflage or aposematic warnings [22,30,31,32]. Defensive venoms, particularly in fish, have evolved to cause immediate and intense pain to the predator, which creates a window of escape time [2]. Defensive venoms have therefore been shaped by selective pressures exerted by predators.

Conversely, venoms used for predation have a benefit of a high energy reward for a successful predatory hunt. Predation is a common driver for venom evolution [2], one that has been studied the most. Predatory venoms have evolved primarily to subdue prey rather than to outright kill them. This is probably because the energetic demands of using venom to kill prey would outweigh the fitness benefits gained over just subduing prey [33,34]. Predatory venoms, unlike defensive venoms, have generally not evolved to cause pain but to immobilise prey [2]. They target neuromuscular pathways to cause paralysis or disrupt hemostasis and the coagulation/anticoagulation of blood [2]. It is the need of evolving toxins to efficiently immobilise prey and the evolution of toxin resistance in prey that drives predatory venom evolution [35].

It should be noted that venoms, in particular predatory venoms, can have both predatory and defensive roles [36,37]. This has led to the evolution of dual purpose venoms [5,38,39]. This would suggest these have arisen despite different, or possibly competing, selective pressures acting on them.

#### 2.1.1. Evolving Venom for Defense in Fish

Many species of fish utilise spines as a form of defence from predatory attacks [12,13]. However, not all spine defences contain venom [13], and having non-venomous spines may not be as effective against predators [40]. Non-venomous spines differ in their defensive strength depending on shape, size, and stoutness, etc., and adding venom increases this defensive strength [40,41]. Thus, antagonistic interactions have led to the evolution of spines and then secondarily to the evolution of venom or to both simultaneously. Yet, if and how spines and venom have evolved in conjunction, which has recently been demonstrated for the venom and venom apparatus of rattlesnakes [42,43], is a topic that needs more investigation.

It has been hypothesised that venom glands in fish evolved by the thickening and aggregation of epidermal cells that produced antiparasitic toxins near defensive spines [44]. Skin secretions in fish contain ichthyocrinotoxins, which are known for having antimicrobial, antiparasitic, and antifouling activities [44,45,46,47,48]. Moreover, experiments on gobies suggest that ichthyocrinotoxins can play an important role in predator avoidance [49,50]. Compounds that are thought to be primarily involved in host–parasite and antimicrobial interactions may therefore have played a role in the evolution of fish venoms, as skin mucus toxins contribute to envenomation effects [20,51,52,53]. Further, evidence suggests that the stonustoxin (SNTX) gene family has evolved from an ancient antiviral protein superfamily [48]. A secondary use of venom for antiparasitic defence has evolved in other taxa as well, such as social Hymenoptera [54] and slow loris [55]. Toxins are spread over the body in slow loris and nests in hymenopterans, reducing parasitic infection [54,55]. This hypothesis of fish venom evolving from skin secretions is plausible, as crinotoxic gobies are more effective at avoiding predation than closely related non-toxic species that are protected solely by spines and tough scales [49], and species of porchthyine toadfish can cause envenomations, yet there is no macroscopic evidence of any form of venom gland associated with spines [12,13,56]. This suggests the toxins may be on the epidermal surface of the spines or located in primitive secretory cells. Similar spine functions can also be seen in other taxa, such as recently discovered venomous frogs (*Corythomantis greeningi* and *Aparasphenodon brunoi*) [57]. They have small skull spines, located near epidermal skin glands filled with toxins. The spines are used to deliver the toxins into the predator’s system through a wound [57]. Frogs, like fish, also have antimicrobial skin toxins [58,59], and it is possible that these venomous frogs have convergently evolved a venom system reminiscent of that found in venomous fish. A recent study has confirmed that mucosal skin secretions and venom extracts in *Scorpaena plumieri* share similar proteins and that these are found across multiple species as well, even in non-venomous fish [20]. However, the authors only attempted to show that skin mucus proteins do not produce any physiological effects different from venom gland envenomations. Their assays did not consider whether skin mucus proteins may be for parasitic defense or might have different biological activities compared to venom gland proteins.

There is little research into predator–prey interactions as drivers of defensive venom evolution in fish. The only available investigations of predator responses to venomous fish prey are focused on catfish [41], and studies of the pharmacological effects of fish venom toxins are restricted to mammal species that are not natural fish predators. One major difficulty is that venomous fish tend to have multiple predators, e.g., stonefish (*Synancea spp*.) are predated on by sharks, rays, and sea snakes [60], whilst (in their natural habitat range) lionfish (*Pterois spp*.) are predated on by sharks, eels, and groupers [60,61]. These different predators may have different susceptibilities to the defensive venom of their prey. Another problem is that defensive venoms are effective against a wide range of organisms, even non-natural predators. Therefore, it is uncertain that a single predator species would drive the evolution of a defensive venom. This taxonomic diversity of predators could explain why many defensive venoms are non-target specific in both venom composition and delivering systems [11,12,13]. Although interactions between predators and venomous fish prey have not been thoroughly investigated, there is strong evidence that such interactions have led to the evolution of defensive spines [62], which are a necessary apparatus for delivering venom.

Based on these ideas it is possible that in some species of fish, host–parasite or antimicrobial interactions may have set the stage for skin toxins to evolve whilst predator–prey interactions have led to the evolution of spines. Selection pressures for increased antipredator defenses then allowed for the recruitment of skin toxins into spine-associated venom. Gene duplication is already thought to have been involved in the evolution of venom in Scorpaeniformes [48,63]. These are hypotheses that need further testing and although antagonistic coevolution may have played a role in evolving spine defenses, the number of convergent origins of fish venom systems suggests that there are strong selection pressures for the evolution of venom in fish.

In contrast to venomous spines, *Meiacanthus* blennies utilise unique venom fangs that are located on the lower jaw [17]. In most fanged venom systems, defense is used as a secondary function after predation. *Meiacanthus’* diets mostly consist of coral polyps, zooplankton, and small invertebrates [64]. Catching this food does not require venom and therefore the venomous fangs suggest a defensive role. However, some research tested the defensive pain inducing toxins of *Meiacanthus* venom with regard to mammalian subjects, which produced no significant reaction [17]. Yet, it is unlikely their venom has evolved to target mammals, and thus pain inducing toxins could still be present but rather target predatory fish. This same study also highlights other toxins for defense causing hypotensive and inflammatory effects. Further, these biochemical assays suggest a defensive role may not be the only ecological function of the venom [17]. Competition is a possible evolutionary driver of fangblenny venom as well (see Section 2.1.3).

#### 2.1.2. Evolving Venom for Predation in Fish

Predation is a strong driver of venom evolution. As previously mentioned, selection pressures acting on venom specificity to prey are thought to arise in many predator–prey interactions. A constant battle between increasing efficiency for subduing prey and toxin resistance of prey lead to these coevolutionary cycles, yet this is an area of little research and may be more complex than simple antagonistic coevolution [26,35].

The use of venom for predation is mostly associated with delivery apparatuses located close to the mouth of an organism, e.g., fangs, pincers, beaks, and probosces [2]. Only two fish taxa utilise fangs/teeth for venom-based feeding, *Monognathus* (jawed eels) and lampreys (Petromyzonidae) [12,17,65]. In jawed eels, the fixed rostral fang is located on the upper jaw [12] (Figure 2). This is highly indicative of the fang playing a role in subduing prey, like in snakes, although the diet of *Monognathus* eels is not well studied. Due to the scarcity of prey items in deep-sea habitats, most deep-sea organisms are opportunistic hunters of many prey types [66]. Consequently, venom for predation would be highly adaptive in desolate environments to ensure prey do not escape. Research on the composition and bioactivity of *Monognathus* venom is needed, as well as observations of how they use their venom.

Lampreys are an ancient lineage of jawless fish that utilise a toothed sucking-buccal cavity. Most species of lamprey engage in parasitic micropredation, attaching their mouth parts to larger hosts in order to blood feed for a prolonged period [65]. This kind of predation has evolved frequently in hematophagous organisms such as leeches, ticks, mosquitos, and bats [67,68,69]. Some studies have highlighted key components of their venom secretions, including anticoagulants, and their nociceptor and immune response inhibitors [14,70,71]. Lampreys are known for their wide dispersal and habitat ranges, being carried to new areas by attaching to migrating hosts [72,73,74,75]. The use of host attachment and blood feeding toxins might have evolved in parallel. The longer a lamprey can attach to a host to reach a specific destination whilst being able to feed, the better its chances of survival throughout the journey. This unique lifestyle may have driven the evolution of this venom system, firstly, by evolving numbing toxins to avoid detection by their hosts and, secondly, by evolving anticoagulants to keep blood flowing over long migration ranges [14,71,74]. Very little research has been conducted on how these micropredatory venoms have evolved, be it before or after the evolution of host-attachment. Lampreys are an ideal model for these investigations as they are the only fish to utilise this strategy.

#### 2.1.3. Evolving Venom for Competition in Fish

Venom used for competition is a rare occurrence with few animals, such as the platypus (*Ornithorhynchus anatinus*) and slow loris (*Nycticebus spp*.) utilising this function. It is worth noting that there is a fine distinction between defensive and competitive venom, as both are interchangeable in function. The distinction between them is based on the selective pressures that elicit the venom evolution and how they are used, be it toward conspecifics or predators.

In fish, it is possible that competition has driven the evolution of one venom system associated with *Meiacanthus* fangblennies. The biological activity of *Meiacanthus grammistes* venom is unique. The venom causes hypotensive, neurotoxic, and proinflammatory effects. These functions act to disorient attackers, rendering coordination and swimming difficult [17]. Yet, their venom may also play an important role in intra- and interspecific competition. Blennies are known for their intense competition for territory and aggressive combat with competitors [76,77,78]. The biological activity of their venom would be advantageous against competitors as disorientation and hinderance in coordination would increase the probability of the competitor becoming an easy target for predators, permanently removing the competition from the environment.

This intense competition for territory may have increased selection pressures on blennies to evolve fangs and venom. The evolution of venom has led to an increasing evolution of mimicry in blennies (see Section 3.2), further suggesting that the function of this trait is highly successful with many mimetic adaptations arising as a consequence [17].

It is uncertain if other fish species also utilise venom in this way. However, it has been suggested that the venom apparatus of stingrays has evolved primarily for a role in intraspecific aggressive encounters [79]. The reasons for this hypothesis are the relatively low toxicity and low metabolic cost of the venom, the use of the sting on conspecific individuals, and observations that the sting is not used on attacking predators. Although defensive stingray barbs injure many people worldwide every year, it has been noted that when handling them, they generally sting very reluctantly [79,80]. The primary role of the relatively non-damaging venom may therefore be in social interactions. However, further research investigating the evolutionary ecology of stingray and fangblenny venoms is clearly needed. This can help us understand the selection pressures arising from competitive and non-predatory aggressive interactions and their possible role in the evolution of these venom systems.

## 3. Consequences of Evolving A Venom System

### 3.1. Aposematism

Aposematic warning signals are prominent in many chemically defended organisms [81]. They display warning signals as bright contrasting and conspicuous patterns, warning predators of their chemical arsenal [62,81]. This kind of defence goes hand-in-hand with defensive venom evolution, and this is no exception for venomous fish. These aposematic patterns have evolved to deter predators in the primary stages of predation (see Section 2) [22]. Evolving warning colouration allows predators to learn avoidance of specific colour patterns, preventing them from being envenomed defensively.

**Figure 3:**
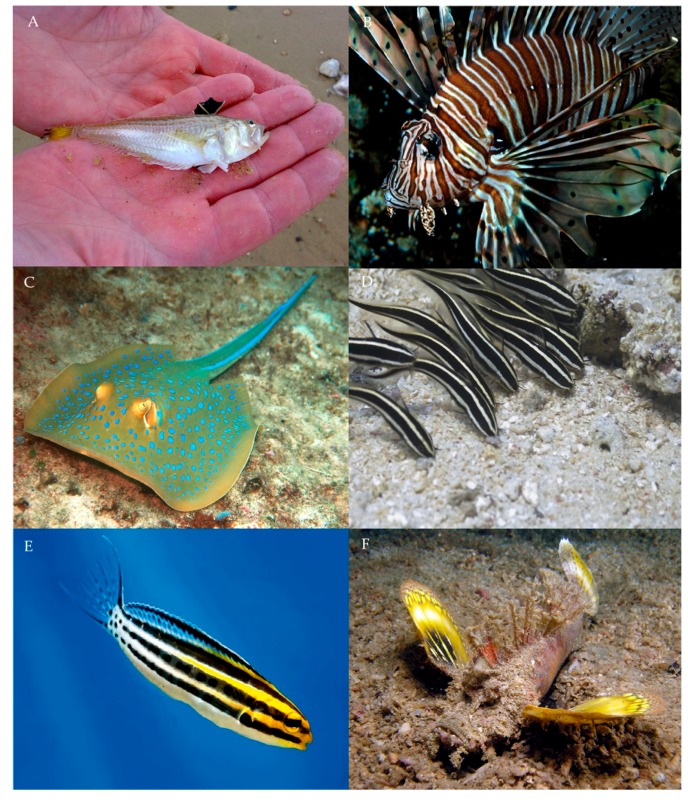
Examples of aposematic colouration adopted by venomous fish species: (**A**) Lesser weever fish (*Echiichthys vipera*); (**B**) Lionfish (*Pterois volitans*); (**C**) Bluespotted ribbontail ray (*Taeniura lymma*); (**D**) Striped eel catfish (*Plotosus lineatus*); (**E**) Striped fang blenny (*Meiacanthus grammistes*); and (**F**) Devil scorpionfish (*Inimicus didactylus*). Image copyrights ©: Rachel Scott, Niels Sloth via www.biopix.dk, Jens Petersen via https://www.en.wikipedia.org CC BY 2.5, Elias Levy via https://www.flickr.com CC BY 2.0, Neil Hepworth—Bauer Media via https://practicalfishkeeping.co.uk, David Harasti via https://www.daveharasti.com.

Since venom has convergently evolved in fish, aposematic colouration has also convergently evolved, as some examples will show. The lesser weever fish (*Echiichthys vipera*) has a dark dorsal fin with yellow spines that stands out in stark contrast to the white/silver colouration of the body (Figure 3A). Contrastingly, dark spine defences highlight to any predator the consequence of attack [62,81]. Weever fish often flare their dorsal spines as a response to nearby movement [82]. This is consistent with the behaviour observed in many other taxa with defensive spines [83]. *E. vipera* also has a yellow and black banding pattern on its caudal fin (Figure 3A): alternating black and yellow colouration is an aposematic pattern that has evolved convergently in a range of venomous and poisonous taxa, including snakes, amphibians, myriapods, spiders, and many hymenopteran species [31,84,85,86,87,88]. Banding patterns may simultaneously act as dazzle camouflage, causing disruptive blurring of the body outline whilst in motion [87]. This causes predators to misjudge the distance and movement of prey, increasing the probability of missing their intended target [89,90]. Lionfish (*Pterois volitans*) are known for their banding pattern (Figure 3B). Because they hunt in the open water, their pattern may not only act as an aposematic warning but also provide active camouflage when hunting and being hunted [81,87]. *Plotosus* catfish and *Meiacanthus* blennies exhibit these banding patterns for both warning and camouflage. (Figure 3D,E). The bluespotted ribbontail ray (*Taeniura lymma*) has a distinctive aposematic spot pattern with iridescent blue colours (Figure 3C). Spot patterns are highly effective in warning predators of a chemical arsenal [91]. Blue spot patterns are efficient in the aquatic environment and are similarly found in such species as blue-ringed octopus (*Hapalochlaena lunulata*) [92]. Many marine predators, such as fish, crustaceans, cetaceans, pinnipeds, and marine birds have visual systems that are sensitive to blue-green wavelength colourations (~400–500nm) [93,94,95,96]. Species of the *Inimicus* genus of scorpionfish have contrasting coloured pectoral and caudal fins in comparison to their body (Figure 3F). These warning fins are flared out when a predator is close, advertising its venom defence.

Because aposematism deters predators at the primary stages of predation, its evolution may be driven by the energetic demands of venom production and replenishment (see Section 4). Venom is energetically costly and many organisms opt to use as little as possible [34,97]. Warning colouration allows organisms to conserve venom by warning predators of their toxins. However, aposematism can evolve in species with non-venomous spines. Thus, energy costs of toxin production may not be the only driver of aposematism.

Aposematic signalling tends to be rare. Data from amphibians suggest that one explanation for this is that over evolutionary time, aposematism is often short lived in lineages with cryptic colouration frequently replacing it [98]. Transition rates from aposematic lineages to either polymorphic or cryptic lineages are substantially higher than in the reverse direction. However, selection for crypsis over aposematism might depend on the strength of the secondary defense [99]. In this case, highly effective venom toxins may allow for aposematism to be maintained, yet weak venom may allow selection pressures to favour crypsis.

Although aposematism is well studied, there are large gaps in the literature that need to be investigated. The selection pressures that act to maintain aposematism or push toward crypsis is an area that should be investigated more. Fish are excellent models as many venomous species exhibit crypsis and/or aposematic patterns, as shown by the above examples. Furthermore, most research has focussed on the implications of prey evolving aposematism. Although there has been much research on the effects of aposematism on predators, more research is needed. Firstly, not much is known about how predators make adaptive decisions in learning avoidance of aposematic signals [100]. Secondly, there are few ideas about how these avoidance behaviours are adopted socially throughout a predator community and how individual predators make adaptive decisions when targeting prey. Much of the work conducted focuses on terrestrial organisms. Venomous fish provide a great platform for comparison between terrestrial and marine taxa. Focussing on the ecology and macroevolution of aposematism in venomous fish may aid in understanding aposematic evolution and how predators learn avoidance.

### 3.2. Mimicry

Mimicry is the evolution of analogous appearances, behaviour, or scent that predators or prey associate with species they either try to lure or avoid, respectively [62]. The purpose of a mimic is to cheat a predator/prey via means of deception, by causing them to be perceived as either a harmful species (Batesian and Müllerian) or a harmless species (aggressive and Mertensian) [62,101,102]. Mimicry can be deemed as parasitic toward the model or mutualistic with both benefitting simultaneously [62]. Many venomous species that are aposematic tend to serve as desirable mimetic models for non-venomous species [17,30,103,104].

#### 3.2.1. Batesian

Batesian mimicry is when an unpalatable species (model) displays aposematic signals which are copied by a palatable species (mimic). In the case of fish, we refer to venom as being the unpalatable trait.

Batesian mimicry has been demonstrated for both poisonous [105] and venomous fish [17]. A recent study found strong evidence for Batesian and aggressive (gaining access to an otherwise non-approachable prey) mimetic phenotypes in fangblennies (Nemophini). There are five genera in Nemophini: one venomous genus (*Meiacanthus*) and four non-venomous genera (*Petroscirtes*, *Cheilodiperus*, *Plagiotremus*, and *Escenius*) [17]. It was found that all non-venomous genera were Batesian mimics of *Meiacanthus*. This Batesian mimicry allows for mimics to appear harmful to predators, falsely warning of their supposed “venom” [17]. It may also act to deceive even closely related species in territorial competition. The *Plagiotremus* genus also shows aggressive mimicry, in that they utilise these colour patterns to gain access to skin feeding in larger fish that only allow specific cleaner species to skin feed [17,106,107]. The phenotypic convergence of colouration between *Meiacanthus* and *Plagiotremus* is one of very few examples of Batesian-aggressive mimicry. It seems that both defence and micropredation may have facilitated this unique convergence [17].

Under normal circumstances, both mimicry systems allow advantage to be taken of the model (*Meiacanthus*). For Batesian mimicry, the model bears all the pressure in educating predators, whilst the mimic gains the benefit of an increased fitness when the mimic population numbers are less than the model [62]. In aggressive mimicry, if the frequency of attacks by the mimic is increased, then the model suffers an increased intolerance by the larger fish and attack rates are increased for the model too [108]. However, when both mimicry systems work in tandem, they are beneficial for both the model and mimic. For example, aggressive attacks on predators by the mimic may strengthen learned avoidance for the model–mimic colouration by predators [106]. When these mimetic systems work together, they may even be categorised as Müllerian with both species being unpalatable or as quasi-Batesian where both species are unpalatable, but the model is more so [109,110,111].

#### 3.2.2. Müllerian

**Figure 4:**
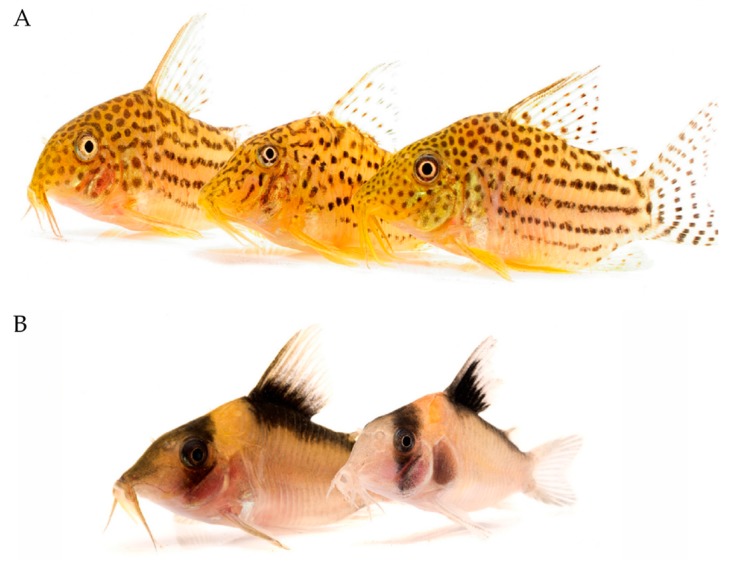
Examples of Müllerian mimetic patterns in venomous Corydoras catfish mimicry ring communities. (**A**) *Corydoras multimaculatus*, *C. araguaiaensis* and *C. sp*. (left to right) and (**B**) *C. imitator* (left) and *C. sp*. (right). Images courtesy of © Martin Taylor via https://www.flickr.com/photos/99775901@N03/.

Müllerian mimicry occurs when two or more chemically defended species share similar colouration [62]. Müllerian mimics share predator education and thus can mutually coexist as a single community structure [62,109], and their aposematism can be maintained over evolutionary time through coevolution of the Müllerian mimics [112]. The abundance of multiple Müllerian mimics in a community can lead to the complex divergence of “mimicry rings” [113,114]. These structures have been observed in venomous Corydoradinae catfish [103]. *Corydoras* mimicry rings are unique in that species within them differ in colouration from related species in distant communities, whilst unrelated species share similar colourations [103] (Figure 4). Alexandrou et al. [103] observed 52 species that adopted 24 mimicry ring communities, with multiple unrelated species all coexisting in stable communities. This has allowed the existence of co-mimics that would be competing for trophic resources without mimicry [103]. Although their patterns are similar, they differ in other morphological features, such as snout length, which lessens competition for resources [103,112].

Research has mostly focussed on the negative aspects of Batesian mimicry, such as how the mimic benefits whilst the model is burdened with predator education. In contrast, positive interactions (e.g., mutualisms) of Müllerian mimicry and even Batesian-aggressive mimicry, which facilitate the longevity and coexistence of multiple species, have been relatively overlooked. Research into multiple mimetic systems acting in conjunction is also lacking. There is an absence of data regarding these systems, whereby two distinct selection pressures act upon multiple species that exhibit mimetic colouration. Research concerning mimicry ring formations is also essential. Understanding how these ring communities diverge and change and the factors influencing the longevity of these systems are poorly understood. Further, investigating venom variation or strength in Corydoradinae mimicry rings will help us understand more complex phenomena such as super-Müllerian mimicry, when two chemically defended mimics have differing toxin strengths, and how these lead to greater learned avoidance by predators [115].

Better understanding of mimetic relationships in fish could highlight key facets of how evolving a venom may affect the ecology in a community structure. This may also help us to understand other mimetic systems, both terrestrial and marine.

## 4. Energetic Implications of Evolving A Venom System

The utilisation of a defensive venom can be costly to an organism’s fitness when the predatory presence is low. This is because the production of toxins is energetically demanding, competing with the energetic demands of growth and reproduction [62]. Therefore, the selective pressures on venom must be balanced by sufficient predatory presence and energy harmonising.

Replenishment of toxins after use can be energetically demanding to an organism [33,97], although not all venoms are metabolically costly [79]. The energy used on replenishment can reduce fitness and survivability, as it can take up to several days [116,117], leaving them chemically undefended. It was shown that venom replenishment of key toxins in *Synanceia horrida* can take 28 days, with full venom yield taking longer depending on feeding conditions [118]. These energetic constraints may potentially explain why many venomous organisms adopt other forms of defence, such as crypsis and aposematism (see Section 3.1) [62]. In venomous fish, although aposematism and crypsis are common, the adoption of a sedentary lifestyle is also prevalent [44]. This is seen in species such as stonefish, scorpionfish, waspfish, and weeverfish. Sedentary fish have low metabolic rates and decreased locomotory functions after feeding, in comparison to active fish [119]. This sedentary/sit-and-wait lifestyle may have evolved in venomous fish for energy conservation, balancing the energetic demands of venom with growth and reproduction. There are certainly many venomous taxa with a sit-and-wait lifestyle, such as viperid snakes and spiders. It is possible that energetic demands of venom have led to the evolution of sedentary lifestyles, as well as aposematic and cryptic colourations.

Venomous taxa can employ other strategies that reduce the energetic costs of venom use, such as venom metering and dry-bites/stings [34]. Venom metering controls the amount of venom injected per bite or sting, gauged by factors such as prey size or predator threat [34,120,121]. Although venom metering has not been investigated in fish, it is unlikely for their defensive venoms. This is based on the morphology of venom structures [12,13]. Venomous spines/barbs lack structures that allow behavioural venom metering provided by fangs or stingers in other venomous taxa. Venoms from spines are released via a pressure mechanism [12,13], whereby the venom gland/tissue is deformed or ruptured upon pressure. It would be difficult for this mechanism to allow control of the venom volume released. However, it is entirely possible that fish that utilise venomous fangs, e.g., *Meiacanthus* and *Monognathus*, could control venom output.

The evidence that venomous taxa can control the volume of venom delivered, along with a sedentary lifestyle in many venomous species, suggests that the energetic cost of venom production is an important constraint both evolutionarily and ecologically. More research is required to assess the energy demands of evolving venom, particularly the ecological constraints that it poses and the adaptations that might help to balance the energy budget.

## 5. Intersexual Variation in Venom

Sexual variation of venom composition has been documented for a few taxa, principally spiders, scorpions, snakes, and a species of fish [122,123,124,125]. Research on *Thalassophyrne maculosa* (cano toadfish) showed that male venom had double the protein content of that of females and differing bioactivities, with males having a greater target affinity to nociceptors whilst females had greater proteolytic activity [123]. From an ecological perspective, one may speculate that these differences could relate to reproduction and brooding. A lower protein concentration in females may be linked to energetic balancing of venom and reproduction. Having a lower yield means energy on venom maintenance can be reallocated to other needs, such as reproduction. Conversely males may have a higher protein yield and potency for guarding of the eggs from predators and conspecifics [126,127]. Similar observations have been made in other species, such as spiders. One study found that females that were carrying eggs had a lower venom yield and fewer proteins present than females without eggs [128]. Yet, venom may not be sex specific but related to other factors such as diet, health, and size. Certainly, sexual dimorphism of size is common in nature, and it could be argued that size is a major factor in explaining why venom potency and yield are different between sexes. However, this does not explain the variance in protein content and bioactivities between sexes.

More research is needed on interspecific venom variation and how this relates to ecology. These differences may be vital in understanding how energy is balanced between venom production and other ecological demands.

## 6. Ecological Niches, Life History, and Venom Evolution

As antipredator defences evolve to reduce predation, it would logically suggest that evolving venom would reduce ecological constraints. For example, an increase in niche space may occur, as less predators would allow for a greater freedom of movement [129].

Research conducted on chemically defended musteloid mammals (they secrete repellent compounds) suggests that chemical defence increases niche space, increasing foraging times, diet diversity, and activity periods [130]. However, no investigations regarding niche space of venomous organisms have been conducted. Since the overarching function of fish venom is for defence, they may similarly increase niche space. A study on butterflyfish (Chaetodontidae) showed that robust and longer dorsal spines led to an increase in riskier foraging behaviour [131]. Although it is unknown if butterflyfish are venomous, their spine morphology has the characteristics of venom spines. Still, it is interesting that defensive spines can alter foraging strategies regardless of whether they deliver venom or not.

Lionfish, *Pterois miles* and *P. volitans*, are known for having broad niches on native reefs, particularly regarding foraging ranges and diet [132,133,134]. This broad niche range is possibly attributable to their venomous spines, with few predators to limit foraging strategies. This might be why they have caused invasive devastation across reefs in the Caribbean and Gulf of Mexico [132,133,134]. Studies comparing the behaviour of native and invasive lionfish populations indicate no difference between foraging strategies and niche ranges, although invaders capture larger sized prey [134]. Is their broad niche range and wide variety of prey items a consequence of their possession of venom on both native and invaded reefs? With little research investigating the ecological implications of venom on foraging ranges and diet, it is difficult to determine. Yet, chemical defence is one aspect in which foraging in new areas without natural predators has led to widespread upheaval of communities [132,133,135]. Lionfish and their invasive behaviour would be an ideal model to understand how niches are affected by the evolution of a defensive venom.

This niche alteration may also occur with predatory venoms. Hematophagous lampreys have a large range of habitats, wide reproductive dispersal and low selectivity of host–prey [74]. It is their lifestyle of host attachment in combination with anticoagulant venom (see Section 2.1.2) that may have allowed for long migrations and dispersal. The evolution of venom certainly allows for lamprey to attach to hosts longer, increasing migration and dispersal, whilst finding new breeding grounds and hosts when resources change. Therefore, it is plausible the evolution of a predatory venom may have aided their successful increase in niche space, range, and host prey. Certainly, more research needs to be conducted investigating how the evolution of both defensive and predatory venoms may alter niche characteristics.

Although much research has shown how venom can vary with changes in prey type [36,136,137], there are fewer investigations about how specific diets may put evolutionary constraints on venom. For example, a shift in diet may allow for constraints of venom evolution to be removed. This concept might fit with the evolution of venom in *Meiacanthus* fangblennies. The evolution of elongated canines evolved at the base of the Nemophini clade; however, venom evolution did not occur until much later with the origin of the *Meiacanthus* genus [17]. Interestingly, *Meiacanthus* is the only genus within Nemophini that has a different diet, tending to feed on small invertebrates [138], rather than opting for scales/skin feeding of larger fish. This is a very interesting facet, as defensive venom might not evolve in skin feeders as the larger fish would feel the effects of venom and therefore learn avoidance of these fish. It has been observed that a dietary shift can lead to the loss of a venom trait as well [139,140]. Therefore, the reverse should also occur, where a change in diet either frees constraints or initiates positive selection pressure for evolving a venom. It is interesting that other factors such as specific diet may play an important role in restricting the evolution of venom when a potential delivery system has already evolved.

Some research has suggested that the evolution of aposematism with regards to a toxic arsenal can also have similar effects on niche [130,141]. Yet, there is a fine divide between discerning if these changes in behaviour occur due to the evolution of the toxins or the warning colouration as both evolve in conjunction with each other. Thus, experiments may be difficult to conduct regarding this aspect.

It is not just the ecological niche that can be affected by the evolution of a chemical defence, life history traits are prone to alteration as well. Chemical defences have evolved to increase longevity by reducing predatory mortality rates [142]. They have been shown to lead to slower life histories and increased longevity in amphibians [143], with venomous fish also corroborating this [144]. Yet, some research contradicts this positive relationship, with findings of a negative correlation in chemically defended musteloid mammals [130]. The authors suggest that physiological mechanisms of chemical defence may reduce life span due to oxidative stress damaging vital tissues shortening life span [145]. They also note that this negative relationship may be due to variables affecting both chemical defence and longevity making them appear correlated [130]. It should be noted the possible contradictions may be due to the difference in chemical defence type and utilisation. It is possible that the chemical defences of musteloid animals (secretions from anal glands) have different ecological consequences for longevity than skin secretions or venoms and this may cause these contrasting patterns.

Future investigations should aim to compare how venom affects the longevity of an organism. Venomous fish can be a perfect model to investigate this, as within families, there are closely related venomous and non-venomous species that can provide a comparison of longevity and venom. Further, investigations into how different functions of chemical defences can lead to differences in longevity, e.g., venom vs. poison or defensive vs. predatory venom functions.

## 7. Diversification Rates and Venom Evolution

The study of ecological factors may illuminate how diversification works and can help us understand how these factors may lead to phenotypic diversity and speciation. The “escape and radiate” hypothesis [129] identifies chemical defences as a factor increasing diversification and speciation. However, very little research has been conducted on this concerning venom [146].

The only study regarding venom diversification in fish showed that venom gland evolution has increased diversification in *Meiacanthus* blennies [147]. Further studies investigating macroevolutionary diversification showed that evolving venom in reptiles and mammals has increased diversification rates as well [3]. Despite this, other research has shown a negative association between diversification and venomous stings in ants [148]. With these differing trends between taxa, the association of diversification and defensive toxins might not be based on the chemical arsenal but the delivery system involved, with some delivery systems being more effective than others. This may be a key area for future investigations.

A study of amphibian chemical defense showed that lineages that were chemically defended had increased speciation rates yet also increased extinction rates [98]. This extinction risk may also play an ecological role in venomous fish lineages. It is noteworthy that most toxic amphibians utilise defensive toxins as poisons rather than venom like fish; however the functional strategy remains the same.

Understanding diversification across lineages using phylogenetic comparative methods such as ancestral state estimations, trait evolution models, convergent evolution and dynamic diversification analyses may provide substantial evidence about the ecological relevance of evolving toxic traits [146]. Fish may provide an excellent model to study these due to the wide array of convergently evolved venoms and large phylogenetic diversity within fishes [12,13]. Further research should aim to demonstrate if diversification rates differ based on the type of venom apparatus evolved. Fish have adopted an array of different apparatus across lineages and thus provide a solid basis for the comparison of diversification. It remains unclear if this increased extinction risk also applies to species that primarily utilise defensive venoms. Fish would be good candidates to investigate this as many venomous taxa are considered threatened species.

## 8. Conclusions and Future Directions

Research regarding the evolutionary ecology of venom systems is scarce, not just in fish but across animals in general. As fish represent the largest group of vertebrates, coupled with their vast convergence of venom, it stands to reason that exploring the evolutionary ecology of venom in fish has great potential to shed light on unanswered questions regarding venom evolution.

Exploring the evolution of defensive fish venom, including its role in interactions with predators, could shed more light on the relationship between skin mucus ichthyocrinotoxins and venom components, as well as illuminate how venom systems have evolved as integrated phenotypes in fish. This could help improve our understanding of how venom systems evolve from systems with other systemic functions, and how predator–prey/host–parasite interactions influence venom evolution.

More studies on how defensive fish venom systems interact with predators are needed to shed more light on the ecological drivers in venom evolution and the mechanisms acting as selection pressures in fish communities. Further investigations on the coevolutionary interactions between venomous fish and their predators can illuminate the relative importance and frequency of different types of ecological and evolutionary dynamics, such as arms races or phenotype matching between predators and prey, in the shaping of venom systems more generally. The convergent evolution of defensive fish venom systems is especially useful for studies that aim to understand how these interactions act to increase divergence and speciation.

Aposematism and mimicry are prevalent consequences of evolving a venom system. Fish highlight these strategies in myriad ways not only from the perspective of phenotypes but also from that of the complex community interactions that arise with mimetic relationships. This makes venomous fish a solid model for the study of these systems.

Applying phylogenetic comparative methods to venomous fish taxa has the potential to highlight how important evolving this system is from an ecological perspective. Understanding how a trait is evolved on a broad scale may further lead to the recognition of how and why these complex systems evolve across longer evolutionary timescales. Furthermore, the link between diversification and extinction [98] urgently needs more research.

By studying the evolutionary ecology of venom, we can begin to cross disciplinary lines within toxinology more confidently. Understanding how venom has evolved on an ecological scale will allow us to better understand the purpose and function of venom on a biochemical and genetic level. Venom systems are integrated phenotypes that comprise many components with interrelated functions across different levels of organisation, from individual toxic molecules to complex morphological delivery systems and from behavioural adaptations to aposematic and mimetic colours and patterns. The study of such systems in large groups such as fish is a promising avenue to advance our understanding of venomous taxa and their evolution.

## References

[1] Fry B.G., Roelants K., Champagne D.E., Scheib H., Tyndall J.D., King G.F., Nevalainen T.J., Norman J.A., Lewis R.J., Norton R.S. (2009). The toxicogenomic multiverse: Convergent recruitment of proteins into animal venoms. Ann. Rev. Genom. Human Genet..

[2] Casewell N.R., Wüster W., Vonk F.J., Harrison R.A., Fry B.G. (2013). Complex cocktails: The evolutionary novelty of venoms. Trends Ecol. Evol..

[3] Harris R.J., Arbuckle K. (2016). Tempo and mode of the evolution of venom and poison in tetrapods. Toxins.

[4] Dutertre S., Jin A.-h., Kaas Q., Jones A., Alewood P.F., Lewis R.J. (2013). Deep venomics reveals the mechanism for expanded peptide diversity in cone snail venom. Mol. Cell. Proteom..

[5] Dutertre S., Jin A.-H., Vetter I., Hamilton B., Sunagar K., Lavergne V., Dutertre V., Fry B.G., Antunes A., Venter D.J. (2014). Evolution of separate predation-and defence-evoked venoms in carnivorous cone snails. Nat. Commun..

[6] Vetter I., J Lewis R. (2012). Therapeutic potential of cone snail venom peptides (conopeptides). Curr. Top. Med. Chem..

[7] Fry B.G., Vidal N., Norman J.A., Vonk F.J., Scheib H., Ramjan S.F.R., Kuruppu S., Fung K., Blair Hedges S., Richardson M.K. (2005). Early evolution of the venom system in lizards and snakes. Nature.

[8] Clark A.M. (1996). Natural Products as a Resource for New Drugs. Pharm. Res..

[9] Fernandez J.H., Neshich G., Camargo A.C.M. (2004). Using bradykinin-potentiating peptide structures to develop new antihypertensive drugs. Genet. Mol. Res..

[10] Triplitt C., Chiquette E. (2006). Exenatide: From the Gila monster to the pharmacy. J. Am. Pharm. Assoc..

[11] Ziegman R., Alewood P. (2015). Bioactive components in fish venoms. Toxins.

[12] Smith W.L., Stern J.H., Girard M.G., Davis M.P. (2016). Evolution of Venomous Cartilaginous and Ray-Finned Fishes.

[13] Smith W.L., Wheeler W.C. (2006). Venom evolution widespread in fishes: A phylogenetic road map for the bioprospecting of piscine venoms. J. Hered..

[14] Li B., Gou M., Han J., Yuan X., Li Y., Li T., Jiang Q., Xiao R., Li Q. (2018). Proteomic analysis of buccal gland secretion from fasting and feeding lampreys (Lampetra morii). Proteome Sci..

[15] Wright J.J. (2009). Diversity, phylogenetic distribution, and origins of venomous catfishes. BMC Evol. Biol..

[16] Revell L.J. (2012). phytools: An R package for phylogenetic comparative biology (and other things). Methods Ecol. Evol..

[17] Casewell N.R., Visser J.C., Baumann K., Dobson J., Han H., Kuruppu S., Morgan M., Romilio A., Weisbecker V., Mardon K. (2017). The evolution of fangs, venom, and mimicry systems in blenny fishes. Curr. Biol..

[18] Baumann K., Casewell N.R., Ali S.A., Jackson T.N., Vetter I., Dobson J.S., Cutmore S.C., Nouwens A., Lavergne V., Fry B.G. (2014). A ray of venom: Combined proteomic and transcriptomic investigation of fish venom composition using barb tissue from the blue-spotted stingray (Neotrygon kuhlii). J. Proteom..

[19] Han H., Baumann K., Casewell N.R., Ali S.A., Dobson J., Koludarov I., Debono J., Cutmore S.C., Rajapakse N.W., Jackson T.N. (2017). The cardiovascular and neurotoxic effects of the venoms of six bony and cartilaginous fish species. Toxins.

[20] Borges M.H., Andrich F., Lemos P.H., Soares T.G., Menezes T.N., Campos F.V., Neves L.X., Castro-Borges W., Figueiredo S.G. (2018). Combined proteomic and functional analysis reveals rich sources of protein diversity in skin mucus and venom from the Scorpaena plumieri fish. J. Proteom..

[21] Malacarne P.F., Menezes T.N., Martins C.W., Naumann G.B., Gomes H.L., Pires R.G., Figueiredo S.G., Campos F.V. (2018). Advances in the characterization of the Scorpaena plumieri cytolytic toxin (Sp-CTx). Toxicon.

[22] Endler J.A., Feder M.E., Lauder G.V. (1986). Defense against predators. Predator-Prey Relationships: Perspectives and Approaches from the Study in Lower Vertebrates.

[23] Endler J.A. (1991). Interactions between predator and prey. Behav. Ecol..

[24] Dawkins R., Krebs J.R. (1979). Arms races between and within species. Proc. R. Soc. Lond. B.

[25] Van Valen L. (1973). A new evolutionary law. Evol. Theory.

[26] Holding M.L., Biardi J.E., Gibbs H.L. (2016). Coevolution of venom function and venom resistance in a rattlesnake predator and its squirrel prey. Proc. R. Soc. B.

[27] Margres M.J., Wray K.P., Hassinger A.T.B., Ward M.J., McGivern J.J., Moriarty Lemmon E., Lemmon A.R., Rokyta D.R. (2017). Quantity, Not Quality: Rapid Adaptation in a Polygenic Trait Proceeded Exclusively through Expression Differentiation. Mol. Biol. Evol..

[28] Brodie E., Formanowicz D., Brodie E. (1991). Predator avoidance and antipredator mechanisms: Distinct pathways to survival. Ethol. Ecol. Evol..

[29] Nelsen D.R., Nisani Z., Cooper A.M., Fox G.A., Gren E.C., Corbit A.G., Hayes W.K. (2014). Poisons, toxungens, and venoms: Redefining and classifying toxic biological secretions and the organisms that employ them. Biol. Rev..

[30] Wüster W., Allum C.S., Bjargardóttir I.B., Bailey K.L., Dawson K.J., Guenioui J., Lewis J., McGurk J., Moore A.G., Niskanen M. (2004). Do aposematism and Batesian mimicry require bright colours? A test, using European viper markings. Proc. R. Soc. Lond. B Biol. Sci..

[31] Mappes J., Marples N., Endler J.A. (2005). The complex business of survival by aposematism. Trends Ecol. Evol..

[32] Speed M.P., Ruxton G.D. (2005). Warning displays in spiny animals: One (more) evolutionary route to aposematism. Evolution.

[33] McCue M.D. (2006). Cost of producing venom in three North American pitviper species. Copeia.

[34] Morgenstern D., King G.F. (2013). The venom optimization hypothesis revisited. Toxicon.

[35] Arbuckle K., de la Vega R.C.R., Casewell N.R. (2017). Coevolution takes the sting out of it: Evolutionary biology and mechanisms of toxin resistance in animals. Toxicon.

[36] Barlow A., Pook C.E., Harrison R.A., Wüster W. (2009). Coevolution of diet and prey-specific venom activity supports the role of selection in snake venom evolution. Proc. R. Soc. Lond. B Biol. Sci..

[37] Boyer L., Alagón A., Fry B., Jackson T., Sunagar K., Chippaux J. (2015). Signs, symptoms and treatment of envenomation. Venom. Reptil. Their Toxins Evol. Pathophysiol. Biodiscovery.

[38] Inceoglu B., Lango J., Jing J., Chen L., Doymaz F., Pessah I.N., Hammock B.D. (2003). One scorpion, two venoms: Prevenom of Parabuthus transvaalicus acts as an alternative type of venom with distinct mechanism of action. Proc. Natl. Acad. Sci. USA.

[39] Whittington C.M., Papenfuss A.T., Bansal P., Torres A.M., Wong E.S., Deakin J.E., Graves T., Alsop A., Schatzkamer K., Kremitzki C. (2008). Defensins and the convergent evolution of platypus and reptile venom genes. Genome Res..

[40] Sismour E.N., Nellis S.C., Newton S.H., Mays D.A., Fine M.L. (2013). An experimental study of consumption of channel catfish Ictalurus punctatus by largemouth bass Micropterus salmoides when alternative prey are available. Copeia.

[41] Wright J.J. (2012). Adaptive significance of venom glands in the tadpole madtom Noturus gyrinus (Siluriformes: Ictaluridae). J. Exp. Biol..

[42] Margres M.J., Wray K.P., Seavy M., McGivern J.J., Sanader D., Rokyta D.R. (2015). Phenotypic integration in the feeding system of the eastern diamondback rattlesnake (Crotalus adamanteus). Mol. Ecol..

[43] Strickland J.L., Smith C.F., Mason A.J., Schield D.R., Borja M., Castañeda-Gaytán G., Spencer C.L., Smith L.L., Trápaga A., Bouzid N.M. (2018). Evidence for divergent patterns of local selection driving venom variation in Mojave Rattlesnakes (Crotalus scutulatus). Sci. Rep..

[44] Cameron A.M., Endean R. (1973). Epidermal secretions and the evolution of venom glands in fishes. Toxicon.

[45] Colorni A., Ullal A., Heinisch G., Noga E. (2008). Activity of the antimicrobial polypeptide piscidin 2 against fish ectoparasites. J. Fish Dis..

[46] Rajanbabu V., Chen J.-Y. (2011). Applications of antimicrobial peptides from fish and perspectives for the future. Peptides.

[47] Ángeles Esteban M. (2012). An overview of the immunological defenses in fish skin. ISRN Immunol..

[48] Ellisdon A.M., Reboul C.F., Panjikar S., Huynh K., Oellig C.A., Winter K.L., Dunstone M.A., Hodgson W.C., Seymour J., Dearden P.K. (2015). Stonefish toxin defines an ancient branch of the perforin-like superfamily. Proc. Natl. Acad. Sci. USA.

[49] Gratzer B., Millesi E., Walzl M., Herler J. (2015). Skin toxins in coral-associated G obiodon species (T eleostei: G obiidae) affect predator preference and prey survival. Mar. Ecol..

[50] Schubert M., Munday P.L., Caley M.J., Jones G.P., Llewellyn L.E. (2003). The toxicity of skin secretions from coral-dwelling gobies and their potential role as a predator deterrent. Environ. Biol. Fishes.

[51] Junqueira M.E.P., Grund L.Z., Orii N.M., Saraiva T.C., de Magalhães Lopes C.A., Lima C., Lopes-Ferreira M. (2007). Analysis of the inflammatory reaction induced by the catfish (Cathorops spixii) venoms. Toxicon.

[52] Monteiro-dos-Santos J., Conceição K., Seibert C.S., Marques E.E., Silva P.I., Soares A.B., Lima C., Lopes-Ferreira M. (2011). Studies on pharmacological properties of mucus and sting venom of Potamotrygon cf. henlei. Int. Immunopharmacol..

[53] Ramos A.D., Conceição K., Silva P.I., Richardson M., Lima C., Lopes-Ferreira M. (2012). Specialization of the sting venom and skin mucus of Cathorops spixii reveals functional diversification of the toxins. Toxicon.

[54] Baracchi D., Francese S., Turillazzi S. (2011). Beyond the antipredatory defence: Honey bee venom function as a component of social immunity. Toxicon.

[55] Grow N.B., Nekaris K. (2015). Does toxic defence in Nycticebus spp. relate to ectoparasites? The lethal effects of slow loris venom on arthropods. Toxicon.

[56] Lopes-Ferreira M., Ramos A.D., Martins I.A., Lima C., Conceição K., Haddad V. (2014). Clinical manifestations and experimental studies on the spine extract of the toadfish Porichthys porosissimus. Toxicon.

[57] Jared C., Mailho-Fontana P.L., Antoniazzi M.M., Mendes V.A., Barbaro K.C., Rodrigues M.T., Brodie E.D. (2015). Venomous Frogs Use Heads as Weapons. Curr. Biol..

[58] Mangoni M.L., Rinaldi A.C., Di Giulio A., Mignogna G., Bozzi A., Barra D., Simmaco M. (2000). Structure–function relationships of temporins, small antimicrobialpeptides from amphibian skin. Eur. J. Biochem..

[59] Rinaldi A.C. (2002). Antimicrobial peptides from amphibian skin: An expanding scenario: Commentary. Curr. Opin. Chem. Biol..

[60] Paxton J.R., Eschmeyer W.N., Kirshner D. (1998). Encyclopedia of Fishes.

[61] Mumby P.J., Harborne A.R., Brumbaugh D.R. (2011). Grouper as a natural biocontrol of invasive lionfish. PLoS ONE.

[62] Ruxton G.D., Sherratt T.N., Speed M.P., Speed M.P., Speed M. (2004). Avoiding Attack: The Evolutionary Ecology of Crypsis, Warning Signals and Mimicry.

[63] Chuang P.-S., Shiao J.-C. (2014). Toxin gene determination and evolution in scorpaenoid fish. Toxicon.

[64] Hundt P.J., Nakamura Y., Yamaoka K. (2014). Diet of combtooth blennies (Blenniidae) in Kochi and Okinawa, Japan. Ichthyol. Res..

[65] Baxter E.W. (1956). Observations on the Buccal Glands of Lampreys (Petromyzonidae). Proc. Zool. Soc. Lond..

[66] Dayton P., Hessler R. (1972). Role of Biological Disturbance in Maintaining Diversity in the Deep Sea.

[67] Ribeiro J. (1987). Role of saliva in blood-feeding by arthropods. Ann. Rev. Entomol..

[68] Apitz-Castro R., Beguin S., Tablante A., Bartoli F., Holt J.C., Hemker H.C. (1995). Purification and partial characterization of draculin, the anticoagulant factor present in the saliva of vampire bats (Desmodus rotundus). Thromb. Haemost..

[69] Francischetti I.M. (2010). Platelet aggregation inhibitors from hematophagous animals. Toxicon.

[70] Gage S.H., Gage-Day M. (1927). The anti-coagulating action of the secretion of the buccal glands of the lampreys (Petromyzon, Lampetra and Entosphenus). Science.

[71] Ito N., Mita M., Takahashi Y., Matsushima A., Watanabe Y.G., Hirano S., Odani S. (2007). Novel cysteine-rich secretory protein in the buccal gland secretion of the parasitic lamprey, Lethenteron japonicum. Biochem. Biophys. Res. Commun..

[72] Meckley T.D., Wagner C.M., Gurarie E. (2014). Coastal movements of migrating sea lamprey (Petromyzon marinus) in response to a partial pheromone added to river water: Implications for management of invasive populations. Can. J. Fish. Aquat. Sci..

[73] Waldman J., Grunwald C., Wirgin I. (2008). Sea lamprey Petromyzon marinus: An exception to the rule of homing in anadromous fishes. Biol. Lett..

[74] Silva S., Araújo M.J., Bao M., Mucientes G., Cobo F. (2014). The haematophagous feeding stage of anadromous populations of sea lamprey Petromyzon marinus: Low host selectivity and wide range of habitats. Hydrobiologia.

[75] Bergstedt R.A., Seelye J.G. (1995). Evidence for Lack of Homing by Sea Lampreys. Trans. Am. Fish. Soc..

[76] Buchheim J.R., Hixon M.A. (1992). Competition for shelter holes in the coral-reef fish Acanthemblemaria spinosa Metzelaar. J. Exp. Mar. Biol. Ecol..

[77] Koppel V.H. (1988). Habitat selection and space partitioning among two Mediterranean blenniid species. Mar. Ecol..

[78] Stephens J.S., Johnson R.K., Key G.S., McCosker J.E. (1970). The comparative ecology of three sympatric species of California blennies of the genus Hypsoblennius Gill (Teleostomi, Blenniidae). Ecol. Monogr..

[79] Enzor L., Wilborn R., Bennett W. (2011). Toxicity and metabolic costs of the Atlantic stingray (Dasyatis sabina) venom delivery system in relation to its role in life history. J. Exp. Mar. Biol. Ecol..

[80] Hughes R., Pedersen K., Huskey S. (2018). The kinematics of envenomation by the yellow stingray, Urobatis jamaicensis. Zoomorphology.

[81] Cott H.B. (1940). Adaptive Coloration in Animals.

[82] Lewis D.B. (1976). Studies of the biology of the lesser weever fish Trachinus vipera Cuvier: I. Adaptations to a benthic habit. J. Fish Biol..

[83] Inbar M., Lev-Yadun S. (2005). Conspicuous and aposematic spines in the animal kingdom. Naturwissenschaften.

[84] Kauppinen J., Mappes J. (2003). Why are wasps so intimidating: Field experiments on hunting dragonflies (Odonata: Aeshna grandis). Anim. Behav..

[85] Schuler W., Hesse E. (1985). On the function of warning coloration: A black and yellow pattern inhibits prey-attack by naive domestic chicks. Behav. Ecol. Sociobiol..

[86] Noonan B.P., Comeault A.A. (2009). The role of predator selection on polymorphic aposematic poison frogs. Biol. Lett..

[87] Hoese F., Law E., Rao D., Herberstein M. (2006). Distinctive yellow bands on a sit-and-wait predator: Prey attractant or camouflage?. Behaviour.

[88] Iniesta L.F., Ratton P., Guerra T.J. (2017). Avian predators avoid attacking artificial aposematic millipedes in Brazilian Atlantic Forest. J. Trop. Ecol..

[89] Hughes A.E., Troscianko J., Stevens M. (2014). Motion dazzle and the effects of target patterning on capture success. BMC Evol. Biol..

[90] Stevens M., Searle W.T.L., Seymour J.E., Marshall K.L., Ruxton G.D. (2011). Motion dazzle and camouflage as distinct anti-predator defenses. BMC Biol..

[91] Dolenska M., Nedved O., Vesely P., Tesarova M., Fuchs R. (2009). What constitutes optical warning signals of ladybirds (Coleoptera: Coccinellidae) towards bird predators: Colour, pattern or general look?. Biol. J. Linn. Soc..

[92] Mäthger L.M., Bell G.R., Kuzirian A.M., Allen J.J., Hanlon R.T. (2012). How does the blue-ringed octopus (Hapalochlaena lunulata) flash its blue rings?. J. Exp. Biol..

[93] Cronin T.W., Marshall N.J., Caldwell R.L. (2000). Spectral tuning and the visual ecology of mantis shrimps. Philos. Trans. R. Soc. Lond. B Biol. Sci..

[94] Gačić Z., Damjanović I., Mićković B., Hegediš A., Nikčević M. (2007). Spectral sensitivity of the dogfish shark (Scyliorhinus canicula). Fish Physiol. Biochem..

[95] Hart N.S. (2004). Microspectrophotometry of visual pigments and oil droplets in a marine bird, the wedge-tailed shearwater Puffinus pacificus: Topographic variations in photoreceptor spectral characteristics. J. Exp. Biol..

[96] Levenson D.H., Ponganis P.J., Crognale M.A., Deegan J.F., Dizon A., Jacobs G.H. (2006). Visual pigments of marine carnivores: Pinnipeds, polar bear, and sea otter. J. Comp. Physiolo. A.

[97] Nisani Z., Dunbar S.G., Hayes W.K. (2007). Cost of venom regeneration in Parabuthus transvaalicus (Arachnida: Buthidae). Comp. Biochem. Physiol. Part A Mol. Integr. Physiol..

[98] Arbuckle K., Speed M.P. (2015). Antipredator defenses predict diversification rates. Proc. Natl. Acad. Sci. USA.

[99] Speed M.P., Franks D.W. (2014). Antagonistic evolution in an aposematic predator–prey signaling system. Evolution.

[100] Skelhorn J., Halpin C.G., Rowe C. (2016). Learning about aposematic prey. Behav. Ecol..

[101] Pasteur G. (1982). A classificatory review of mimicry systems. Ann. Rev. Ecol. Syst..

[102] Sheppard P. (1959). The Evolution of Mimicry; a Problem in Ecology and Genetics.

[103] Alexandrou M.A., Oliveira C., Maillard M., McGill R.A., Newton J., Creer S., Taylor M.I. (2011). Competition and phylogeny determine community structure in Müllerian co-mimics. Nature.

[104] Greene H.W., McDiarmid R.W. (1981). Coral snake mimicry: Does it occur?. Science.

[105] Caley J.M., Schluter D. (2003). Predators favour mimicry in a tropical reef fish. Proc. R. Soc. Lond. Ser. B Biol. Sci..

[106] Cheney K.L. (2010). Multiple selective pressures apply to a coral reef fish mimic: A case of Batesian–aggressive mimicry. Proc. R. Soc. Lond. Ser. B Biol. Sci..

[107] Fujisawa M., Sakai Y., Kuwamura T. (2018). Aggressive mimicry of the cleaner wrasse by Aspidontus taeniatus functions mainly for small blennies. Ethology.

[108] Cheney K.L., Côté I.M. (2005). Frequency-dependent success of aggressive mimics in a cleaning symbiosis. Proc. R. Soc. Lond. Ser. B Biol. Sci..

[109] Rowland H.M., Ihalainen E., Lindström L., Mappes J., Speed M.P. (2007). Co-mimics have a mutualistic relationship despite unequal defences. Nature.

[110] Rowland H.M., Mappes J., Ruxton G.D., Speed M.P. (2010). Mimicry between unequally defended prey can be parasitic: Evidence for quasi-Batesian mimicry. Ecol. Lett..

[111] Taylor M.I. (2017). Evolution: Fangtastic Venoms Underpin Parasitic Mimicry. Curr. Biol..

[112] Wright J.J. (2011). Conservative coevolution of Müllerian mimicry in a group of rift lake catfish. Evolution.

[113] Marek P.E., Bond J.E. (2009). A Müllerian mimicry ring in Appalachian millipedes. Proc. Natl. Acad. Sci. USA.

[114] Symula R., Schulte R., Summers K. (2001). Molecular phylogenetic evidence for a mimetic radiation in Peruvian poison frogs supports a Müllerian mimicry hypothesis. Proc. R. Soc. Lond. Ser. B Biol. Sci..

[115] Balogh A.C., Gamberale-Stille G., Leimar O. (2008). Learning and the mimicry spectrum: From quasi-Bates to super-Müller. Anim. Behav..

[116] Currier R.B., Calvete J.J., Sanz L., Harrison R.A., Rowley P.D., Wagstaff S.C. (2012). Unusual stability of messenger RNA in snake venom reveals gene expression dynamics of venom replenishment. PLoS ONE.

[117] Nisani Z., Boskovic D.S., Dunbar S.G., Kelln W., Hayes W.K. (2012). Investigating the chemical profile of regenerated scorpion (Parabuthus transvaalicus) venom in relation to metabolic cost and toxicity. Toxicon.

[118] Saggiomo S.L., Zelenka C., Seymour J. (2017). Relationship between food and venom production in the estuarine stonefish Synanceia horrida. Toxicon.

[119] Fu S.-J., Zeng L.-Q., Li X.-M., Pang X., Cao Z.-D., Peng J.-L., Wang Y.-X. (2009). The behavioural, digestive and metabolic characteristics of fishes with different foraging strategies. J. Exp. Biol..

[120] Nisani Z., Hayes W.K. (2011). Defensive stinging by Parabuthus transvaalicus scorpions: Risk assessment and venom metering. Anim. Behav..

[121] Young B.A., Zahn K. (2001). Venom flow in rattlesnakes: Mechanics and metering. J. Exp. Biol..

[122] Amorim F.G., Costa T.R., Baiwir D., De Pauw E., Quinton L., Sampaio S.V. (2018). Proteopeptidomic, Functional and Immunoreactivity Characterization of Bothrops moojeni Snake Venom: Influence of Snake Gender on Venom Composition. Toxins.

[123] Lopes-Ferreira M., Sosa-Rosales I., Bruni F.M., Ramos A.D., Portaro F.C.V., Conceição K., Lima C. (2016). Analysis of the intersexual variation in Thalassophryne maculosa fish venoms. Toxicon.

[124] Ward M.J., Ellsworth S.A., Hogan M.P., Nystrom G.S., Martinez P., Budhdeo A., Zelaya R., Perez A., Powell B., He H. (2018). Female-biased population divergence in the venom of the Hentz striped scorpion (Centruroides hentzi). Toxicon.

[125] Zobel-Thropp P.A., Bulger E.A., Cordes M.H., Binford G.J., Gillespie R.G., Brewer M.S. (2018). Sexually dimorphic venom proteins in long-jawed orb-weaving spiders (Tetragnatha) comprise novel gene families. PeerJ.

[126] Gross M.R., Sargent R.C. (1985). The evolution of male and female parental care in fishes. Am. Zool..

[127] Hoffman S.G., Robertson D.R. (1983). Foraging and reproduction of two Caribbean reef toadfishes (Batrachoididae). Bull. Mar. Sci..

[128] Herzig V., Ward R.J., dos Santos W.F. (2002). Intersexual variations in the venom of the Brazilian ‘armed’spider Phoneutria nigriventer (Keyserling, 1891). Toxicon.

[129] Ehrlich P.R., Raven P.H. (1964). Butterflies and plants: A study in coevolution. Evolution.

[130] Arbuckle K., Brockhurst M., Speed M.P. (2013). Does chemical defence increase niche space? A phylogenetic comparative analysis of the Musteloidea. Evol. Ecol..

[131] Hodge J.R., Alim C., Bertrand N.G., Lee W., Price S.A., Tran B., Wainwright P.C. (2018). Ecology shapes the evolutionary trade-off between predator avoidance and defence in coral reef butterflyfishes. Ecol. Lett..

[132] Layman C.A., Allgeier J.E. (2012). Characterizing trophic ecology of generalist consumers: A case study of the invasive lionfish in The Bahamas. Mar. Ecol. Prog. Ser..

[133] Betancur-R R., Hines A., Acero P A., Ortí G., Wilbur A.E., Freshwater D.W. (2011). Reconstructing the lionfish invasion: Insights into Greater Caribbean biogeography. J. Biogeogr..

[134] Cure K., Benkwitt C.E., Kindinger T.L., Pickering E.A., Pusack T.J., McIlwain J.L., Hixon M.A. (2012). Comparative behavior of red lionfish Pterois volitans on native Pacific versus invaded Atlantic coral reefs. Mar. Ecol. Prog. Ser..

[135] Phillips B.L., Brown G.P., Webb J.K., Shine R. (2006). Invasion and the evolution of speed in toads. Nature.

[136] Daltry J.C., Wüster W., Thorpe R.S. (1996). Diet and snake venom evolution. Nature.

[137] Prator C.A., Murayama K.M., Schulz J.R. (2014). Venom variation during prey capture by the cone snail, Conus textile. PLoS ONE.

[138] Losey G.S. (1972). Predation protection in the poison-fang blenny, Meiacanthus atrodorsalis, and its mimics, Ecsenius bicolor and Runula laudandus (Blenniidae). Pac. Sci..

[139] Li M., Fry B., Kini R.M. (2005). Eggs-only diet: Its implications for the toxin profile changes and ecology of the marbled sea snake (Aipysurus eydouxii). J. Mol. Evol..

[140] Li M., Fry B.G., Kini R.M. (2005). Putting the brakes on snake venom evolution: The unique molecular evolutionary patterns of Aipysurus eydouxii (Marbled sea snake) phospholipase A2 toxins. Mol. Biol. Evol..

[141] Santos J.C., Cannatella D.C. (2011). Phenotypic integration emerges from aposematism and scale in poison frogs. Proc. Natl. Acad. Sci. USA.

[142] Bosher B.T., Newton S.H., Fine M.L. (2006). The spines of the channel catfish, Ictalurus punctatus, as an anti-predator adaptation: An experimental study. Ethology.

[143] Hossie T., Hassall C., Knee W., Sherratt T. (2013). Species with a chemical defence, but not chemical offence, live longer. J. Evol. Biol..

[144] Blanco M.A., Sherman P.W. (2005). Maximum longevities of chemically protected and non-protected fishes, reptiles, and amphibians support evolutionary hypotheses of aging. Mech. Ageing Dev..

[145] Blount J.D., Speed M.P., Ruxton G.D., Stephens P.A. (2009). Warning displays may function as honest signals of toxicity. Proc. R. Soc. Lond. B Biol. Sci..

[146] Arbuckle K. (2018). Phylogenetic Comparative Methods can Provide Important Insights into the Evolution of Toxic Weaponry. Toxins.

[147] Liu S.-Y.V., Frederich B., Lavoué S., Chang J., Erdmann M.V., Mahardika G.N., Barber P.H. (2018). Buccal venom gland associates with increased of diversification rate in the fang blenny fish Meiacanthus (Blenniidae; Teleostei). Mol. Phylogenet. Evol..

[148] Blanchard B.D., Moreau C.S. (2017). Defensive traits exhibit an evolutionary trade-off and drive diversification in ants. Evolution.

